# Early to Mid-term Results of Coracoclavicular Ligament Reconstruction Using the Infinity-Lock Button System in the Management of Traumatic Acromioclavicular Joint Dislocations and Lateral End of Clavicle Fractures

**DOI:** 10.7759/cureus.60936

**Published:** 2024-05-23

**Authors:** Akhilesh Pradhan, Meraj Akhtar, Ramnadh Pulavarti

**Affiliations:** 1 Trauma and Orthopaedics, United Lincolnshire Hospitals NHS Trust, Lincoln, GBR

**Keywords:** infinity-lock button management, ligament, acromio clavicular joint, clavicle fractures, coracoclavicular reconstruction

## Abstract

Background

The management of distal clavicle injuries with disruption of the coracoclavicular ligaments (CCLs) is challenging. The aim of this study was to assess the functional results of reconstructing the CCLs with the Infinity-Lock Button System using validated outcome measures, patient satisfaction scores, return to pre-injury activity, and complications.

Methods

A total of 28 cases of CCL disruption were assessed over a seven-year period, which included 14 lateral end-of-clavicle fractures and 14 acromioclavicular joint (ACJ) dislocations. All patients underwent stabilisation of the CCLs using the Infinity-Lock Button System. Patients were assessed preoperatively and postoperatively using validated outcome measures such as visual analogue scale (VAS), Oxford Shoulder Score (OSS), Quick Disabilities of Arm, Shoulder & Hand (Q-DASH) scores, return to pre-injury level of activities, patient satisfaction scores, and complications were reviewed.

Results

The mean age of patients was 36.7 years (18-74 years). The mean follow-up was 38.6 months (8-68 months). The mean time to surgery was 75.8 days (3-619 days). There was a statistically significant improvement in postoperative VAS, OSS, and Q-DASH scores compared to the preoperative (p-value <0.001). Out of the 28 participants, 23 (82.1%) returned to pre-injury level of activities, 25 (89.7%) reported ‘good’ or ‘excellent’ outcomes following the intervention, and none of the patients reported functional instability postoperatively. None of the patients required revision surgery or implant removal.

Conclusion

CCL reconstruction is vital in the management of clavicular injuries. This study demonstrates the safe use of the Infinity-Lock Button System in these injuries with statistically significant improvement in early patient-reported outcome measures, return to pre-injury level of activities, and subjective patient satisfaction.

## Introduction

Injuries of the lateral end of the clavicle (LEC) can present as acromioclavicular joint (ACJ) dislocation or displaced fractures of the LEC. These can be painful and disabling, especially in patients involved in high-energy activities/sports. These injuries often result in coracoclavicular ligament (CCL) disruption leading to grade 3 or above traumatic ACJ dislocation or displaced lateral end clavicle fractures with associated instability. Traumatic ACJ dislocations have an approximate incidence of 2-9 per 10,000 individuals while lateral end of clavicle fractures have an incidence of 50 per 100,000 [1,2]. The majority of lateral end of clavicle injuries occur in men with decreasing incidence in both genders with age [3]. The most common mechanism of injury is a direct fall onto the shoulder or direct impact trauma.

The CCLs (conoid and trapezoid ligaments) are essential supports of the LEC. The ACJ is stabilised by the acromioclavicular ligaments which control anteroposterior and superolateral translation whilst the CCLs ensure restraint to inferior and medial translation of the LEC. The CCLs ensure the action of complex shoulder movements without compromising stability by providing a restrictive mechanism to shoulder girdle muscles such as the trapezius, rhomboids, and serratus anterior [4].

The role of anatomical reconstruction of the CCLs in the management of ACJ dislocations and LEC fractures has been widely established within the literature. At the time of initial injury, patients often report pain and an inability to perform activities of daily living (ADLs). In addition, displaced LEC fractures associated with CC disruption have a high rate of non-union and are deemed unstable injuries. For traumatic ACJ dislocations, the Rockwood Classification system has been widely used to determine instability. Patients with Type I and II ACJ injuries deemed as stable injuries, are managed non-operatively. Type III ACJ dislocations are currently managed on a case-to-case basis; however, recent literature does demonstrate superior outcomes with operative management [5]. Patients with Rockwood IV, V and VI are characteristically unstable injuries requiring surgical fixation [6]. In a minority of cases of displaced LEC fractures, there is damage to the adjacent deltoid or trapezius muscles with buttonholing of the LEC through the muscle. The deltoid and trapezius muscles contribute to the dynamic stability of the LEC and ACJ, therefore this could lead to instability, loss of terminal degrees of motion, and loss of function. 

A wide array of surgical options exist which have evolved with time including autograft or allograft reconstruction, e.g., use of semitendinosus or tibialis anterior or fixation with suture button devices [7-9]. More than 150 surgical techniques have been described in the literature regarding operative management of ACJ dislocations or lateral clavicle fracture fixation/reconstruction with CC disruption. Recently, there has been an increasing trend towards anatomical reconstruction of the CCLs. Treatment options can be divided into four discrete groups: (i) synthetic ligaments and suture loop fixation e.g., GORE-TEX® (Gore Medical, Newark, Delaware, United States), TightRope® (Arthrex, Naples, Florida, United States), (ii) the Infinity-Lock system (Neoligaments Ltd, Leeds, West Yorkshire, United Kingdom) allograft or autograft reconstruction, e.g., hamstring autograft, (iii) ligament or tendon transfer, e.g., Weaver-Dunn procedure, and (iv) fixation with metalwork e.g., Bosworth screw, hook plate fixation. Prior methods of stabilisation have involved screw fixation (the Bosworth screw), use of Kirschner wires (modified Phemister procedure), and tension band wiring techniques but these have fallen out of favour in some units due to metalwork failure or prominence as well as loss of reduction associated with these strategies [10].

The use of button or suture fixation does place the patient at increased risk of iatrogenic fracture due to the presence of anchor bone tunnels as well as common postoperative complications such as wound infection, stiffness, chronic pain, and fixation failure. However, synthetic ligaments such as the Infinity-Lock button system have provided an effective method of fixation with satisfactory clinical outcomes reported in the literature [11]. The Infinity-Lock button system (Neoligaments Ltd) is a reliable and reproducible technique for stabilisation of the ACJ in the context of CCL disruption. The system consists of a polyethylene terephthalate tube tape (240 mm x 7 mm) with an eyelet that loops around the coracoid process alongside a titanium alloy button (Ti-6AI-4V) used to secure the clavicle attachment. The benefits of the Infinity-Lock button system include reduced likelihood of iatrogenic fracture due to the lack of requirement of a coracoid bone tunnel for fixation. The system mimics a near anatomical position of the tube tape with the placement of only a single clavicle tunnel in a vertical orientation. Furthermore, the high strength of the tube tape exceeds the natural CCL load which can maintain long-term reduction and eliminate the need for further tissue grafting. As a result of these theoretical advantages, the Infinity-Lock button system was chosen for use within our patient population.

This study aims to characterise validated patient-reported outcome measures after the anatomical fixation of the CCLs in the context of ACJ dislocation or LEC fracture. The objectives of our study were to primarily assess functional outcomes by comparing the pre-injury and post-reconstruction range of motion, using validated measures such as Quick Disabilities of Arm, Shoulder & Hand (Q-DASH) and Oxford Shoulder Score (OSS) to compare outcomes, assessment of patient satisfaction, and early/mid-term review of postoperative complications using the Infinity-Lock button system. There is limited literature regarding the patient experience with the Infinity-Lock button system in these injuries.

## Materials and methods

We prospectively analysed 28 cases of distal clavicle injuries with CCL disruption over a period of seven years. Patients were followed up from a single surgeon’s practice (author RP) at the Lincoln County Hospital, United Lincolnshire Hospitals NHS Trust, Lincoln, United Kingdom between February 2016 and May 2023. The study was approved by the United Lincolnshire Hospitals NHS Trust (approval number: L0822). Inclusion criteria were patients over the age of 18 years who had sustained an LEC or ACJ dislocation with a fully accessible medical and radiological record, including preoperative and postoperative radiographs. Cases included 14 patients with LEC fractures (Neer type II and type V) and 14 patients with traumatic acromioclavicular joint dislocations (Rockwood type III, IV and V).

Displaced LEC fractures were treated with open reduction and internal fixation using plate osteosynthesis in addition to the reconstruction of CCLs using the Infinity-Lock button system. Patients with traumatic ACJ dislocation were treated with CCL reconstruction using the Infinity-Lock button system and, where necessary, additional excision of the LEC. All operations were performed using the technique recommended by the manufacturers of the Infinity-Lock button system (Neoligaments Ltd). All operations were performed by a single consultant orthopaedic surgeon with a subspecialisation in upper limb trauma (author RP) and all outcome measures were conducted by authors AP and RP to ensure standardisation of practice. Patients <18 years, patients with incomplete records, incorrectly coded records, and duplicate data were excluded.

Data collection and analysis

Data collection included patient comorbidities, occupation, hobbies, levels of pre-injury activity, and records of any previous ipsilateral shoulder injuries or surgeries. Patients were assessed preoperatively and at least six months postoperatively using validated outcome measures such as visual analogue scores (VAS), OSS, Q-DASH scores, return to pre-injury level of activities, subjective patient satisfaction, as well as radiological assessment of fracture union. Preoperative and post-reconstructive range of motion was assessed and the ability to conduct full forward flexion, abduction, and internal and external rotation was assessed on both clinic follow-up and final telephonic consultation. All complications were reviewed. Continuous data was expressed as the mean ± SD and categorical data with actual count and percentage unless stated otherwise. Statistical analysis was performed using Spearman’s correlation coefficient, p<0.05 indicates statistical significance.

## Results

A total of 28 patients (14 LEC fractures and 14 traumatic ACJ dislocations) were included in the study. Nineteen (67.9%) patients were male of which 12 (63.2%) had ACJ dislocations, and 10 (35.7%) patients were female of which eight (80%) had LEC fractures and two (20%) had traumatic ACJ dislocations. The mean age of patients in the study was 36.7 years (range: 18-74 years). Nineteen of the 28 patients (67.6%) were non-smokers and none of the patients in the study had either type 1 or type 2 diabetes. 

Of injuries sustained either to the clavicle or ACJ, 26 (92.9%) were isolated and did not involve injury to other bones or soft tissues. Two patients in the ACJ group sustained other injuries; one patient sustained rib fractures which were managed non-operatively while the second patient sustained a simple pneumothorax requiring chest drain insertion as definitive management. Twenty-seven of the 28 patients (96.4%) did not have any prior injury to the ipsilateral shoulder; one patient in the clavicle group had a previous clavicle fracture which had fully healed with non-operative management prior to their current injury.

Of the 28 injuries, 16 (57.1%) were left-sided, and 25 (89.3%) were right-hand dominant individuals. Twenty-five (89.3%) patients were involved in jobs involving moderate or high-demand activities. Table 1 displays the individual demographics for each group. The mean time to surgery was 75.8 days (3-619 days). At the postoperative follow-up, the mean Q-DASH score was 7.2 (range: 0-22, SD 7.3), the mean OSS was 44.1 (range: 33-48, SD 3.4), the mean VAS score was 17.0 (range: 0-40, SD 12.1). Table 2 displays the individual patient-reported outcome measures combined for both groups. There was a statistically significant difference between preoperative and postoperative scores of all patient-reported outcomes, i.e., Q-DASH, VAS, and OSS (p<0.001). No statistically significant difference was noted in patient-reported outcome measures when comparing the two groups at postoperative follow-up (Table 3).

**Table 1:** Demographic and clinical characteristics of patients in the two groups

	Group A: Lateral end clavicle fracture (N=14)	Group B: Acromioclavicular joint dislocation (N=14)
Number of patients and gender	14 (8 female, 6 male)	14 (2 female, 10 male)
Age	19-55 years (mean 36, SD 13.03)	18-74 years (mean 46.8, SD 18.49)
Time to surgery	3-619 days (mean 114.79, SD 172.95)	3-281 days (mean 68.4, SD 93.19)
Length of follow-up	6-68 months (mean 36.5, SD 22.72)	6-77 months (mean 52, SD 23.33)
Pre-injury activities	78.6% high demand or moderate demand (11/14)	92.8% high demand or moderate demand (13/14)
Side of injury	6 (42.8%) non-dominant arm	7 (50%) non-dominant arm

**Table 2:** Preoperative and postoperative patient-reported outcome measures at final follow-up for both groups (combined results) The data has been represented with analysis using Spearman’s correlation coefficient and significance measured as a two-sided p-value, where p<0.05 denotes statistical significance. VAS: visual analogue scale; OSS: Oxford Shoulder Score; Q-DASH: Quick Disabilities of Arm, Shoulder & Hand

Scores	Mean	N	Std. deviation	Std. error mean	Significance (p-value)
Preoperative VAS	66.71	28	9.19	1.73	P<0.001
Postoperative VAS	16.96	28	12.12	2.29	P<0.001
Preoperative OSS	18.50	28	6.56	1.24	P<0.001
Postoperative OSS	44.14	28	3.39	0.64	P<0.001
Preoperative Q-DASH score	69.03	28	17.56	3.32	P<0.001
Postoperative Q-DASH score	7.16	28	7.29	1.37	P<0.001

**Table 3:** Comparison of preoperative and postoperative patient-reported outcome measures for the individual groups The data has been represented with analysis using Spearman’s correlation coefficient and significance measured as a two-sided p-value, where p<0.05 denotes statistical significance. VAS: visual analogue scale; OSS: Oxford Shoulder Score; Q-DASH: Quick Disabilities of Arm, Shoulder & Hand

Scores	Groups	Mean	N	Std. deviation	Std. error mean	Significance (p-value)
Preoperative VAS score	Group 1	66.86	14	10.42	2.79	p=0.94
	Group 2	66.58	14	8.16	8.16	p=0.94
Postoperative VAS score	Group 1	12.50	14	11.89	11.89	p<0.05
	Group 2	21.42	14	10.99	2.94	p<0.05
Preoperative OSS	Group 1	19.58	14	7.70	2.06	p=0.4
	Group 2	17.42	14	5.30	1.41	p=0.4
Postoperative OSS	Group 1	44.14	14	3.71	0.99	p=0.9
	Group 2	44.14	14	3.20	0.85	p=0.9
Preoperative Q-DASH score	Group 1	63.64	14	19.22	5.13	p=0.105
	Group 2	74.42	14	14.45	3.86	p=0.106
Postoperative Q-DASH score	Group 1	7.46	14	8.62	2.3	p=0.83
	Group 2	6.85	14	5.98	1.5	p=0.83

**Figure 1:**
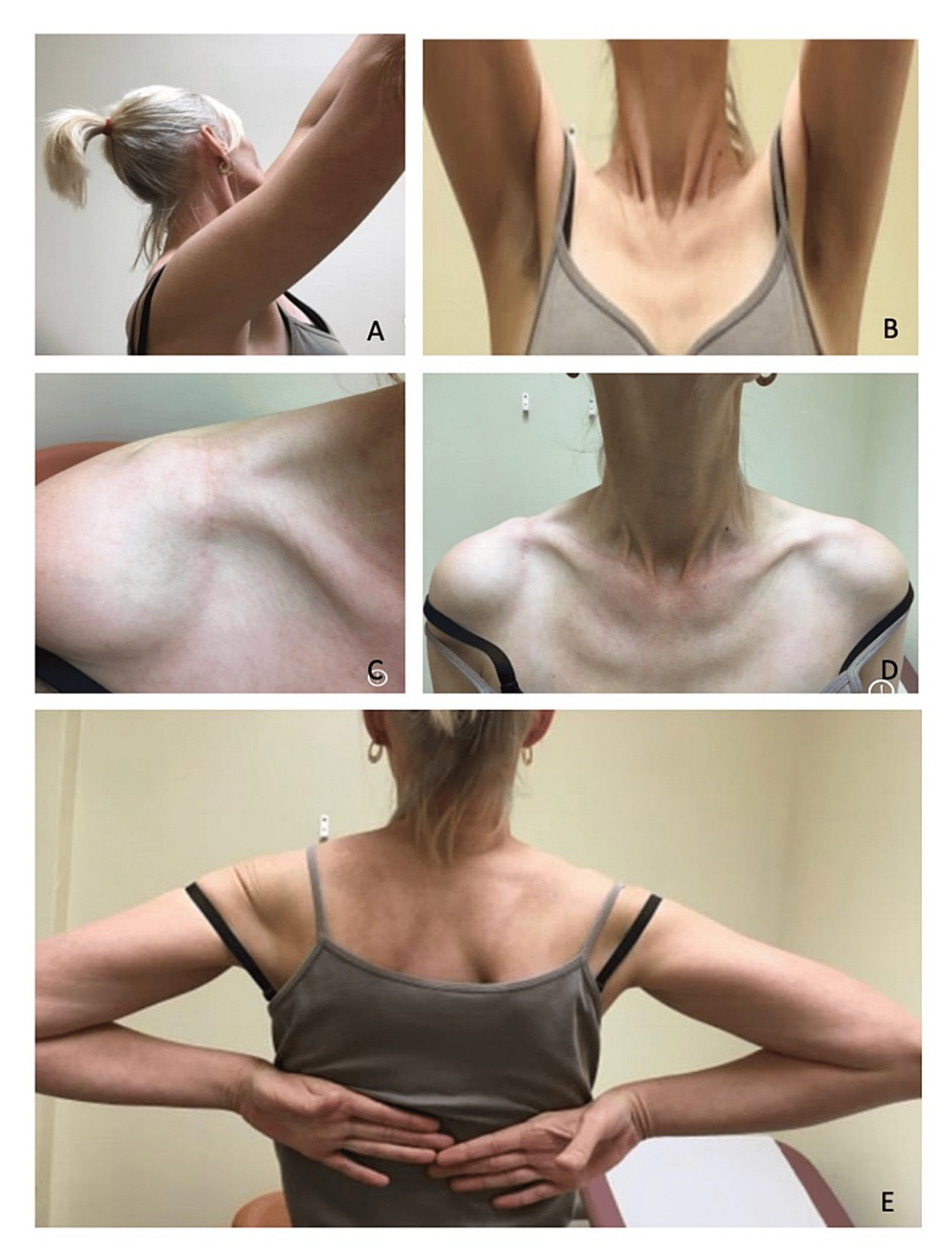
Postoperative range of motion assessed in the final clinic follow-up visit of ACJ dislocation with CCL reconstruction A, B demonstrate full range of forward flexion, C shows a well-healed surgical scar, D demonstrates symmetry of clavicles, and E demonstrates full range of internal rotation ACJ: acromioclavicular joint; CCL: coracoclavicular ligament

**Figure 2:**
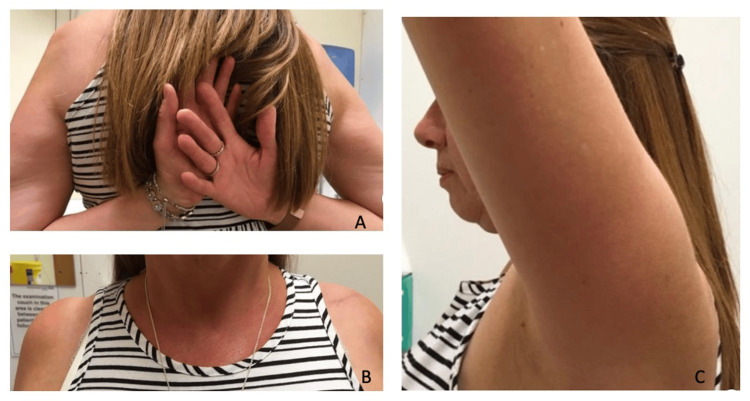
Postoperative range of motion assessed in the final clinic follow-up visit of LEC fracture with CCL reconstruction A demonstrates full range of internal rotation, B demonstrates well-healed surgical scar and clavicle symmetry, and C demonstrates full range of forward flexion LEC: lateral end of clavicle; CCL: coracoclavicular ligament

The mean follow-up in this study was 38.6 months (8-68 months). There were no intraoperative complications in either group. Prior to injury, all patients in both groups had full range of motion (ROM) of their ipsilateral shoulder. None of the patients had symptomatic rotator cuff disease/degenerative joint disease prior to injury. Of the 28 participants, 23 (>80%) patients had full restoration of ipsilateral shoulder ROM post-fixation. In five (17.9%) patients, out of the 28, with some limitations in ROM, this was predominantly described as subjective limitations in abduction and forward flexion movements only. External and internal rotation movements were deemed to be intact upon both clinical assessment and at the final telephonic follow-up. Patients had an average of two postoperative clinic visits for follow-up (range: one to four) and there was only one telephonic follow-up appointment, which was the terminal follow-up for patients between 36-38 months postoperatively. Figures 1-2 demonstrate examples of ROM and scar cosmesis for both ACJ and LEC patients during the final clinic visit.

**Figure 3:**
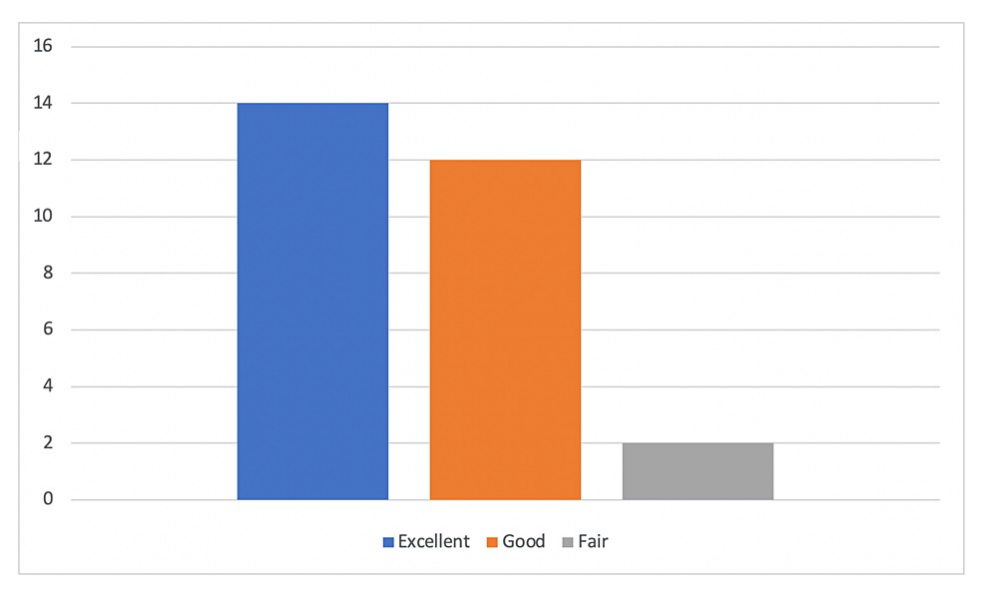
Subjective patient satisfaction score for both groups

Twenty-six (92.9%) patients returned to their pre-injury level of activities and 25 (89.7%) had subjective satisfaction reported as either ‘good’ or ‘excellent’ with the operative intervention; none of the patients reported ‘poor’ or ‘inadequate’ satisfaction scores. Figure 3 demonstrates subjective satisfaction scores reported by patients in both groups combined. None of the patients reported any clinical symptoms or signs of ACJ instability. Satisfactory radiological union was achieved in all cases except one; one case in the LEC group required iliac crest bone grafting which eventually resulted in radiological union and satisfactory clinical presentation at follow-up. No patient required revision surgery for instability; none of the patients underwent implant removal or any further procedures e.g., manipulation of joint or joint injections.

## Discussion

Various surgical techniques have been described for CCL repair/reconstruction in the context of LEC fractures or ACJ dislocation [7-9]. However, there is limited literature analysing the effects of the Infinity-Lock button system on patient-reported outcome measures and overall satisfaction scores. Our study demonstrates the positive impact the use of the Infinity-Lock button system has on patient-reported outcome measures such as the Q-DASH score and OSS. Most patients had full restoration of their pre-injury ROM and were able to accomplish ADLs, hobbies, and occupational work without hindrance.

The Q-DASH questionnaire contains 11 items and measures the patient’s ability to complete tasks and assesses symptom severity. A higher score indicates a greater level of disability in contrast to a lower score reflecting a lower level of disability (0 'no disability' to 100 'most severe disability'). Hence, a Q-DASH score of 7.3 represents an almost normal level of functionality. Similarly, the OSS is a validated tool to assess the degree of pain and severity caused by shoulder pathology. Modified in 2009, the OSS reflects higher values to represent a lower level of disability [12,13]. Our study demonstrates that post-surgical fixation of the CCLs using the Infinity-Lock system has similar results to an asymptomatic population without any upper limb pathology [12]. An average score of 44 emphasises near normal ADLs including dressing, washing, conducting housework and minimal concerns with pain at night time. The absence of pain was also reflected in the VAS score, which was scaled from 0-100, with an average score of 17 demonstrating low levels of pain controlled with simple analgesia such as paracetamol and ibuprofen. None of the patients in the study required regular opioid use postoperatively and this is a testament to alleviation of pain after CCL fixation using Infinity-Lock.

Furthermore, this study demonstrates a high level of subjective patient satisfaction with their overall experience of CCL fixation; more than three-quarters of patients in the study were either satisfied or very satisfied with the care provided and their functional outcomes after the injury, at the time of fixation, and during the postoperative period. There was no statistically significant difference between satisfaction between the LEC and ACJ groups and both groups had high levels of satisfaction. The low levels of complications present in both groups may have also contributed to high levels of satisfaction. Only two patients in the ACJ group had postoperative symptoms of complex regional pain syndrome, which did not require further surgical intervention. This is a relatively common complication present with CC fixation after ACJ dislocation as stated in the literature [14].

This study explores the relationship between CC fixation in the context of LEC fracture or ACJ dislocation and patient-related outcome measures. It identified that the use of the Infinity-Lock button system provides a high level of patient satisfaction with good functional outcomes regarding ADLs and pain management. It provides firm evidence for the safe use of Infinity-Lock in the fixation of the CCLs with a low level of complications and postoperative instability. The average follow-up of 38.6 months demonstrates the lack of severe complications within the early and intermediate postoperative period.

However, there are a few limitations to this study. Even though data was collected prospectively, the number of patients in each group is small. Therefore, to provide more robust evidence, a prospective study with a larger cohort of patients should be conducted in the future. This could include a larger cohort of patients from a single surgeon's practice, such as in this study, to ensure uniformity of surgical technique and its reflection upon patient-related outcome measures. Furthermore, future studies should aim to have a longer period of follow-up to assess for long-term outcomes of implant fixation.

## Conclusions

Our study provides evidence for the safe and reliable use of the Infinity-Lock button system for the fixation of the CCLs in the context of LEC fractures or ACJ dislocations. More than 75% of patients were satisfied with their overall experience with the Infinity-Lock system and patient-related outcome measures were reflective of a high level of activity postoperatively with resumption of ADLs, occupation, ROM, and low levels of postoperative pain. Future studies should focus on prospective data collection with a larger patient cohort to further provide evidence regarding patient-related outcome measures after CCL fixation.
